# Identification and experimental validation of biomarkers associated with mitochondrial and programmed cell death in major depressive disorder

**DOI:** 10.3389/fpsyt.2025.1564380

**Published:** 2025-04-30

**Authors:** Shengjie Xiong, Lixin Liao, Meng Chen, Qing Gan

**Affiliations:** ^1^ Department of Psychiatry, Chengdu Second People’s Hospital, Chengdu, Sichuan, China; ^2^ Department of Obstetrics and Gynecology, Chengdu Second People’s Hospital, Chengdu, Sichuan, China; ^3^ Department of Emergency, Chengdu Second People’s Hospital, Chengdu, Sichuan, China

**Keywords:** major depressive disorder, biomarkers, programmed cell death, mitochondria, bioinformatics analysis

## Abstract

**Background:**

Major depressive disorder (MDD) is associated with mitochondrial dysfunction and programmed cell death (PCD), though the underlying mechanisms remain unclear. This study aimed to investigate the molecular pathways involved in MDD using a transcriptomic analysis approach.

**Methods:**

Transcriptomic data related to MDD were obtained from public databases. Differentially expressed genes (DEGs), PCD-related genes (PCDs), and mitochondrial-related genes (MitoGs) were analyzed to identify key gene sets: PCD-DEGs and MitoG-DEGs. Correlation analysis (|correlation coefficient| > 0.9, p < 0.05) was performed to select candidate genes. Protein-protein interaction (PPI) network analysis and intersection of four algorithms were used to identify key candidate genes. Machine learning and gene expression validation were employed, followed by reverse transcription-quantitative polymerase chain reaction (RT-qPCR) for further validation. A nomogram was developed to predict MDD probability based on biomarkers. Additional analyses included immune infiltration, regulatory networks, and drug predictions.

**Results:**

CD63, IL17RA, and IL1R1 were identified as potential biomarkers, with significantly higher expression levels in the MDD cohort. These findings were validated by RT-qPCR. A nomogram based on these biomarkers demonstrated predictive capacity for MDD. Differential immune cell infiltration was observed, with significant differences in nine immune cell types, including activated T cells and eosinophils, between the MDD and control groups. ATF1 was identified as a common transcription factor for CD63, IL17RA, and IL1R1. Shared miRNAs for CD63 and IL1R1 included hsa-miR-490-3p and hsa-miR-125a-3p. Drug prediction analysis identified 50 potential drugs, including verteporfin, etynodiol, and histamine, targeting these biomarkers.

**Conclusion:**

CD63, IL17RA, and IL1R1 are key biomarkers for MDD, providing insights for diagnostic development and targeted therapies. The predictive nomogram and drug predictions offer valuable tools for MDD management.

## 1 Introduction

Major Depressive Disorder (MDD) is a severe mood disorder characterized by dominant depressive mood, which includes core symptoms such as anhedonia, cognitive and memory impairments, and loss of interest (1). By 2030, MDD is projected to be the leading cause of the global burden of disease (2). Known risk factors for MDD include genetic predisposition, social environment, psychological factors, hormonal imbalances, and neurotransmitter dysregulation (3, 4). Despite various hypotheses, such as the monoamine, cytokine, circadian rhythm, neurotrophic, and inflammation hypotheses (5), a comprehensive understanding of the pathophysiology of MDD remains elusive. In terms of treatment strategy, active initial treatment is the best choice to deal with MDD, and the combination treatment model shows a good application prospect, which can not only improve the response rate of patients, but also help reduce the loss of MDD patients in the course of short - and long-term treatment (6). However, long-term use of antidepressants is associated with a range of side effects, including mood retarding (7), sexual dysfunction (8), and weight gain (9), which further affect patients’ quality of life and treatment compliance. The emergence of biomarkers provides us with more accurate diagnosis and intervention means, which can help reduce the economic health burden of MDD on society, and they provide new possibilities for improving diagnosis methods, optimizing interventions, innovative treatment methods, and predicting treatment response (10). Therefore, it is of vital significance for the research and treatment of MDD to deeply explore the unelucidated physiological and biochemical mechanisms of MDD and actively search for potential biomarkers.

Mitochondria, as the primary energy producers in cells, are central to several physiological functions, including signal transduction, regulation of reactive oxygen species (ROS), substance metabolism, and apoptosis (11). Damage to mitochondrial DNA disrupts cellular connectivity and communication, ultimately compromising cellular function and health (12). Research has highlighted the link between mitochondrial dysfunction and depression, with depressive, melancholic, and anxious states more frequently observed in individuals with mitochondrial impairments (13). Moreover, peroxisome proliferator-activated receptor gamma coactivator 1-alpha (PGC-1α) is a key part of the mitochondrial genome transcription system that maintains mitochondrial biogenesis in the brain (14), and its dysfunction can affect mitochondrial function and neuronal health and is associated with depression (15). Increased mitochondrial fragmentation has been noted in MDD, with the severity of the disorder correlating with alterations in mitochondrial autophagy proteins and mitochondrial dynamics (16). At the same time, it has been found that reactive oxygen species and nitrogen species can cause damage to brain function by regulating the activity of neurotransmitter systems, especially glutamatergic systems, which are closely related to the neurobiology of depression (17). Despite these observations, the specific target proteins involved in the mitochondrial-MDD interaction remain under investigation.

Programmed cell death (PCD) is a regulated, autonomous process by which cells systematically terminate their life activities, a mechanism essential for tissue development and homeostasis in multicellular organisms (18). The PCD gene Pdcd4 has been implicated in depression by specifically inhibiting proteins associated with neuronal function, thus promoting the progression of depressive symptoms. Suppressing Pdcd4 expression has been shown to alleviate these symptoms (19), with recent studies providing direct evidence that targeting Pdcd4 can improve depression outcomes (20). A key pathological feature of MDD is astrocyte damage, particularly through pyroptosis, a form of PCD, in chronic stress-induced depressive models (21). From a cellular biology perspective, PCD contributes to the pathogenesis of MDD by disrupting or impairing neuronal synapses during the disease’s prolonged course (22). Given these observations, the interplay between MDD and PCD warrants further exploration. As previously noted, mitochondrial bioactivity and function are integral to MDD (23), and mitochondrial signaling is closely linked with various PCD mechanisms (24). Previous studies have made progress in the role of mitochondrial and PCD mechanisms in MDD, but did not correlate the two. The current study fills this gap by integrating the mechanism of action of mitochondria and PCD and revealing their synergistic effect and pathophysiological mechanism in MDD, which provides a new direction for deeper understanding of MDD and the development of new treatment strategies.

Physiological and pathological state of the whole body, but also provide rich information for the analysis of immune infiltration. In this study, genes associated with 18 different cell death modes (including necrosis, anoikis, ferroptosis, cup death, lysosome-dependent cell death, and heat death) and MitoGs were included based on peripheral blood samples from public databases. Through differential expression analysis, enrichment analysis, and protein-protein interaction (PPI) network analysis, potential biomarkers for MDD were identified. A nomogram model was then constructed to assess the predictive power of these biomarkers for the disease. Additionally, a range of bioinformatics methods, including molecular regulatory network analysis, immune infiltration analysis, and drug prediction analysis, were employed to offer valuable insights for MDD diagnosis and the development of novel therapeutic strategies. The flow of this study was shown in **Supplementary Figure S1** (25).

## 2 Materials and methods

### 2.1 Data collection

Transcriptome sequencing data were sourced from the Gene Expression Omnibus (GEO) database (https://www.ncbi.nlm.nih.gov/geo/). The training set comprised GSE76826 (GPL17077 platform), which included 10 blood samples from patients with MDD and 12 control blood samples (**Supplementary Table S1**). Depressive state was measured using the Structured Interview Guide for Hamilton Depression (SIGH-D) scale. The validation set, GSE98793 (GPL570 platform), contained 128 MDD blood samples and 64 control blood samples (**Supplementary Table S2**). 64 patients in the MDD patient group were also diagnosed with Generalized Anxiety Disorder based on the results of the MINI questionnaire assessment. A total of 1,548 PCD genes were extracted from the literature (26) (**Supplementary Table S3**), while 1,136 mitochondrial-related genes (MitoGs) were obtained from the MitoCarta3.0 database (http://www.broadinstitute.org/mitocarta) as referenced in the literature (27) (**Supplementary Table S4**).

### 2.2 Identification of candidate genes

Differentially expressed genes (DEGs) in both MDD and control samples within GSE76826 were identified using the limma package (v 3.54.1) (28), with thresholds set at p < 0.05 and |log_2_ fold change (FC)| > 0.5. Subsequently, the ggplot2 package (v 3.3.6) (29) was employed to generate a volcano plot, visually representing these DEGs and highlighting the top 10 upregulated and downregulated genes based on |log_2_FC|. A heatmap displaying the expression patterns of the top 10 DEGs was created using the pheatmap package (v 1.0.12) (30), with gene expression sorted by |log_2_FC|. To identify PCD-related genes exhibiting differential expression, DEGs were intersected with PCDs, yielding PCDs-DEGs. Similarly, DEGs were intersected with MitoGs to derive MitoGs-DEGs. These intersections were visualized using the ggvenn package (v 0.1.9) (31). Spearman correlation analysis, conducted with the psych package (v 2.2.9) (32), assessed the correlation between PCDs-DEGs and MitoGs-DEGs, with a threshold of |correlation coefficient (cor)| > 0.9 and p < 0.05. After eliminating duplicates, genes with significant correlations were selected as candidate genes for further analysis.

### 2.3 Enrichment analysis and protein-protein interaction network of candidate genes

Gene Ontology (GO) and Kyoto Encyclopedia of Genes and Genomes (KEGG) pathway analyses were performed using the clusterProfiler package (v 4.6.2) (33), with p < 0.05 as the significance threshold. The top 15 GO pathways and top 10 KEGG pathways were visualized by ranking them in ascending order of p-values. To explore protein interactions among the candidate genes, a PPI network was constructed using the Search Tool for the Retrieval of Interacting Genes (STRING) database (https://string-db.org/) with a confidence score threshold of 0.4. The cytoHubba plugin in Cytoscape (v 3.9.1) (34) was used to evaluate genes associated with each node, applying algorithms including Degree, Maximum Clique Centrality (MCC), Maximum Neighborhood Component (MNC), and Density of Maximum Neighborhood Component (DMNC). The top 30 genes for each algorithm, based on ranking scores, were selected. An intersection analysis was performed to identify key candidate genes identified by the various algorithms.

### 2.4 Machine learning algorithms and identification of candidate biomarkers

The Boruta algorithm was initially employed to identify Boruta feature genes using the Boruta package (v 8.0.0) (35). The highest importance value of the shadow features (shadow Max) was set as a benchmark, and genes with importance values exceeding this threshold were retained as Boruta feature genes (p = 0.01, mcAdj = T, maxRuns = 300). Next, random forest (RF) analysis was performed on the key candidate genes using the randomForest package (v 4.7-1.1) (36). The number of trees was optimized to minimize errors, avoid overfitting and reduce computational costs, and after re-training the model, RF feature genes were selected from the key candidates based on whether the importance value exceeded the median. Additionally, the key candidate genes underwent rigorous evaluation via the Support Vector Machine-Recursive Feature Elimination (SVM-RFE) algorithm. Key candidate genes were classified using support vector machine (SVM), and the genes with lower importance were ranked according to their importance and gradually eliminated, so that the most relevant genes to the disease were screened out through several iterations. During the evaluation process, 5-fold cross-validation was used to calculate the error rates of different feature sets, and the importance ranking, error rate and accuracy of genes in each iteration were recorded, and the genes corresponding to the feature set with the lowest error rate were finally selected as SVM-RFE feature genes. Finally, the genes selected by the three machine learning algorithms were intersected to identify the candidate biomarkers.

### 2.5 Identification of biomarkers

To assess the expression levels of candidate biomarkers, datasets GSE98793 and GSE76826 were analyzed, and the expression differences between MDD and control groups were compared using the Wilcoxon test. Significant expression differences across both datasets with consistent trends were visualized using a violin plot generated with the ggplot2 package (v 3.3.6), and these genes were designated as biomarkers (p < 0.05). To validate the accuracy of these biomarkers, reverse transcription-quantitative polymerase chain reaction (RT-qPCR) was performed using blood samples from 5 patients with MDD and 5 healthy individuals. All samples were obtained from Chengdu Second People’s Hospital, with informed consent from the donors. The study was approved by the Medical Ethics Review Committee of Chengdu Second People’s Hospital (approval number: [KY]PJ2024246). Total RNA was extracted using the Trizol method (Ambion, 15596018CN, USA) (37), and cDNA was synthesized using the SweScript First Strand cDNA Synthesis Kit (Servicebio, G3333-50, China). GAPDH was used as an internal reference gene, and the expression levels of biomarkers were calculated using the 2^-ΔΔCt^ method (38). Statistical significance was considered at p < 0.05. Primer sequences are listed in **Supplementary Table S5**. The GraphPad Prism (v 8.0) (39) was used for data analysis and visualization.

### 2.6 Construction and evaluation of nomogram

A nomogram was developed using the rms package (v 6.5.0) (40), and the pROC package (v 1.18.0) was used to generate a receiver operating characteristic (ROC) curve to evaluate its predictive performance, with an area under the curve (AUC) > 0.7. Calibration curves were then plotted using the rms package to assess the nomogram’s accuracy. The results indicated no significant difference between the predicted values and actual observations, confirming that the model was well-calibrated (p > 0.05). Furthermore, decision curve analysis (DCA) was performed to evaluate the clinical utility of the nomogram, utilizing the rmda package (v 1.6) (https://github.com/mdbrown/rmda).

### 2.7 Immune infiltration analysis

Immune infiltration analysis provided insights into various immune cell populations, predicted treatment responses, and contributed to the development of immunotherapeutic strategies by revealing the complex interactions between immune cells and biomarkers. Initially, the gene expression profiles of each sample in the GSE76826 dataset were enriched by the ssGSEA algorithm (41) using the set of 28 immune-related genes provided by the TISIDB database (http://cis.hku.hk/TISIDB/) in conjunction with the GSVA software package (v 1.46.0) (42). The ssGSEA algorithm was used to compute each immune cell gene set enrichment scores that reflect the relative abundance or activity of specific immune cell types in the sample. Based on the analysis of these enrichment scores, we can infer the composition and proportions of different immune cell types in the samples, thus further revealing the characteristics of the immune microenvironment. The Wilcoxon test (MDD *vs*. control, p < 0.05) was used to evaluate differences in the proportion of immune cells between MDD and control groups. The results were visualized using the ggplot2 package (v 3.3.6). Differential immune cell infiltration was then visualized using the pheatmap package (v 1.0.12). Furthermore, Spearman correlation analysis was conducted to assess correlations between differential immune cells and between biomarkers and differential immune cells across all samples in GSE76826, using the corrplot package (v 0.92) (43) (|cor| > 0.3, p < 0.05).

### 2.8 Regulatory network analysis

The regulatory network analysis provided a comprehensive mapping of molecular interactions, facilitating the identification of key regulatory hubs, uncovering complex biological pathways, and aiding the prediction of therapeutic targets. Initially, NetworkAnalyst (https://www.networkanalyst.ca/NetworkAnalyst/) was used to identify transcription factors (TFs) associated with the biomarkers. The microcosm database (https://tools4mirs.org/software/mirna_databases/microcosm-targets/) was then used to predict microRNAs (miRNAs) with regulatory roles in relation to the biomarkers. Visualization of the TF-mRNA and miRNA-mRNA regulatory networks was performed using Cytoscape software (v 3.9.1).

### 2.9 Drug prediction

The Drug SIGnatures DataBase (DSigDB) (https://dsigdb.tanlab.org/DSigDBv1.0/) was used to identify potential drugs associated with biomarkers, retaining the top 50 compounds with combined scores ranging from 0.90 to 1,733.47. The drug-mRNA regulatory network was visualized using Cytoscape software (v 3.9.1).

### 2.10 Ethics approval statement

The studies involving human participants were reviewed and approved by the Medical Ethics Review Committee of Chengdu Second People’s Hospital, with written informed consent provided by the patients/participants for their participation in the study.

### 2.11 Statistical analysis

Statistical analysis was performed using the R programming language (v 4.2.2). Group differences were assessed using the Wilcoxon test, with p < 0.05 considered statistically significant. Comparisons between groups in the RT-qPCR analysis were made using t-tests.

## 3 Results

### 3.1 Identification of 55 candidate genes

**Figure 1:**
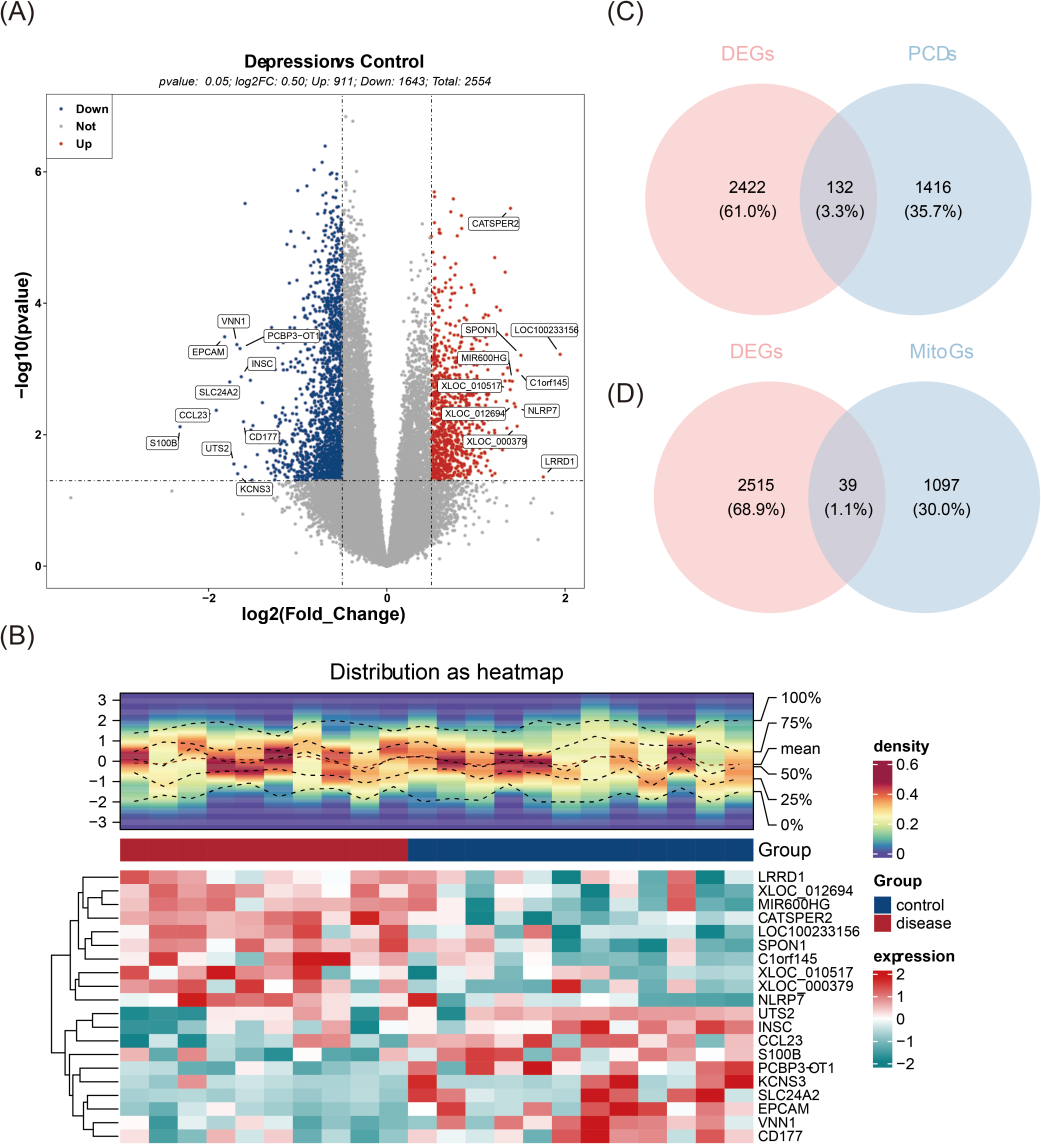
Identification of 55 candidate genes. **(A)** Volcano plot of differentially expressed genes (DEGs), with red dots representing upregulated genes, blue dots representing downregulated genes, and grey dots indicating non-significant genes. **(B)** Heatmap displaying the differential expression of genes, where high expression is indicated in red and low expression in green. The upper section is a heatmap of the expression density of the top10 genes down-regulated on the sample, showing the lines of the five quartiles and the mean; the lower section is a heatmap of the expression of the top10 genes down-regulated on the sample. **(C)** Venn diagram illustrating the intersection of DEGs and PCDs. **(D)** Venn diagram showing the intersection of DEGs and MitoGs.

The differential expression analysis initially revealed a total of 2,554 DEGs, with 911 up-regulated and 1,643 down-regulated in MDD samples compared to controls (**Figures 1A, B**). A total of 132 PCDs-DEGs and 39 MitoGs-DEGs were then identified (**Figures 1C, D**). Spearman correlation analysis further narrowed the selection down to 55 candidate genes for subsequent investigation (**Supplementary Table S6**).

### 3.2 Functional analysis of 55 candidate genes

**Figure 2:**
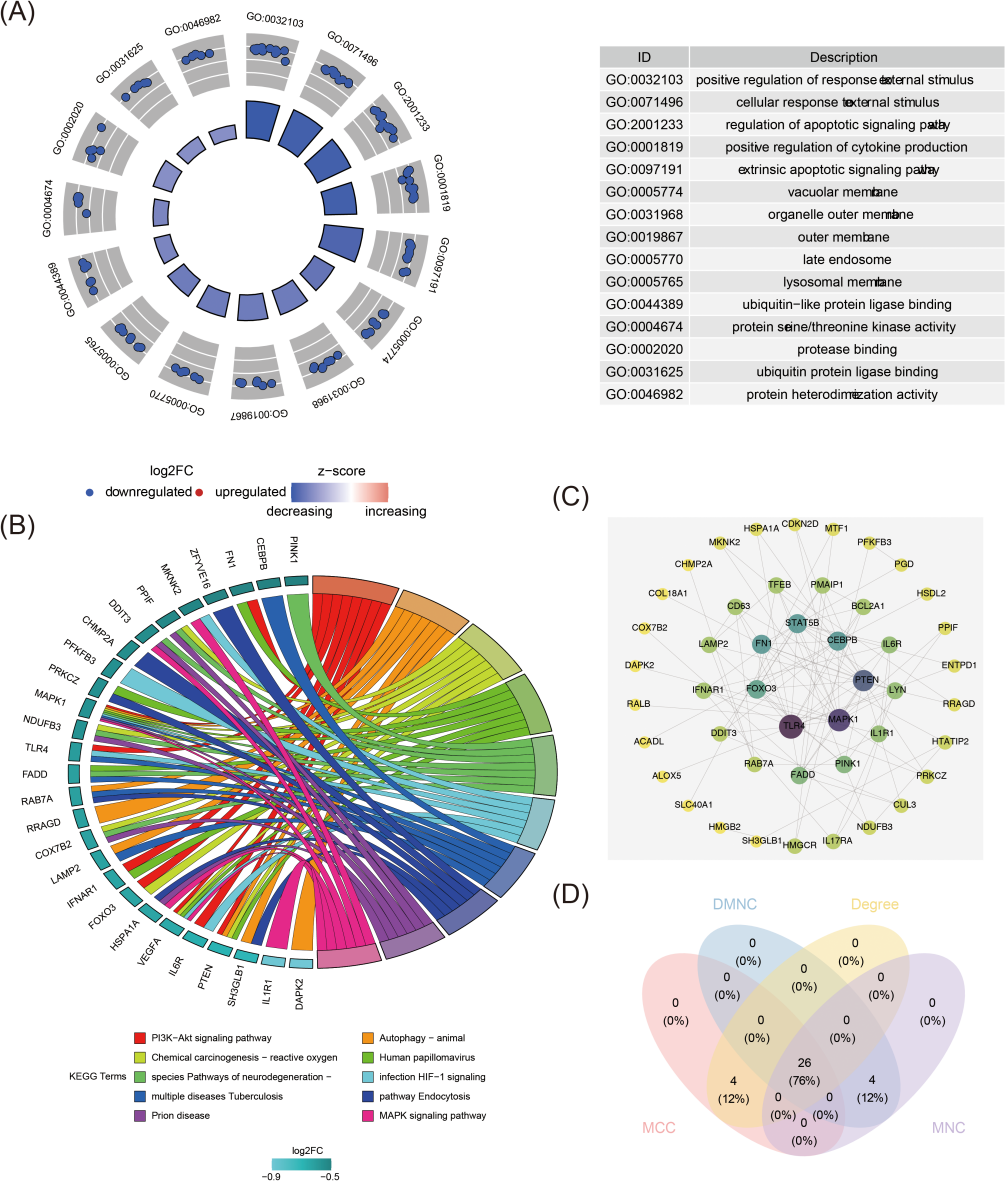
Functional analysis of 55 candidate genes. **(A)** Gene Ontology (GO) enrichment analysis for candidate genes. The figure consists of left and right parts, the left half is the GO enrichment analysis circular graph, the inner circle is the bar graph, the height of the bar graph indicates the significance of the pathway, the higher the more significant; the bar graph is the z-score, the darker the color the bigger the z-score is, the z-score score can imply that the pathway is up-regulated/down-regulated; the outer circle shows a scatterplot of the expression level of the genes in each pathway, the upregulated indicates upregulated genes in each pathway, and downregulated indicates downregulated genes in each pathway. The right half is GO-enriched pathway description information. **(B)** Kyoto Encyclopedia of Genes and Genomes (KEGG) pathway enrichment analysis of candidate genes. The left half of the circle is the name of the enriched genes, the color shade represents the size of log2FC, the darker the color, the larger the log2FC, and the blue is the down-regulated genes; the right half of the circle is the enriched functional pathways, different colors represent different pathways, the pathway names corresponding to the colors can be referred to the Terms at the bottom of the picture, and the size of the color squares change with the number of enriched genes, the more genes are enriched in the pathway, the larger the color squares are. The more genes are enriched in the pathway, the larger the color square is. The middle lines link the genes enriched in different pathways. Some genes may be enriched in more than one pathway at the same time, suggesting that they may have multiple regulatory functions. **(C)** Protein-protein interaction network of candidate genes. Each circle represents a gene, the connecting lines represent the presence of interactions, and the colors represent the size of the degree, with yellow to purple indicating that the degree is small to large. **(D)** Venn diagram of intersecting genes identified by four different algorithms in “CytoHubba”.

Functional enrichment analysis identified key biological processes, pathways, and gene interactions, providing deeper insight into the molecular mechanisms underlying MDD. The candidate genes were significantly enriched across 1,129 GO signaling pathways, including 994 biological processes (BPs) such as the positive regulation of response to external stimuli, 70 cellular components (CCs) such as the vacuolar membrane, and 65 molecular functions (MFs) such as ubiquitin-like protein ligase binding. Additionally, 77 KEGG signaling pathways were identified, including the PI3K-Akt signaling pathway, MAPK signaling pathway, and HIF-1 signaling pathway (**Figures 2A, B**, **Supplementary Tables S7, S8**). The PPI network for the candidate genes contained 46 nodes and 104 edges, with 26 key candidate genes identified from the intersection of four algorithms, including TLR4, PTEN, and MAPK1 (**Figures 2C, D**, **Supplementary Table S9**). Notably, genes such as CEBPB, STAT5B, FN1, TLR4, MAPK1, PTEN, and FOXO3 were positioned centrally within the network, suggesting significant interactions with other proteins.

### 3.3 Identification and expression validation of biomarkers

**Figure 3:**
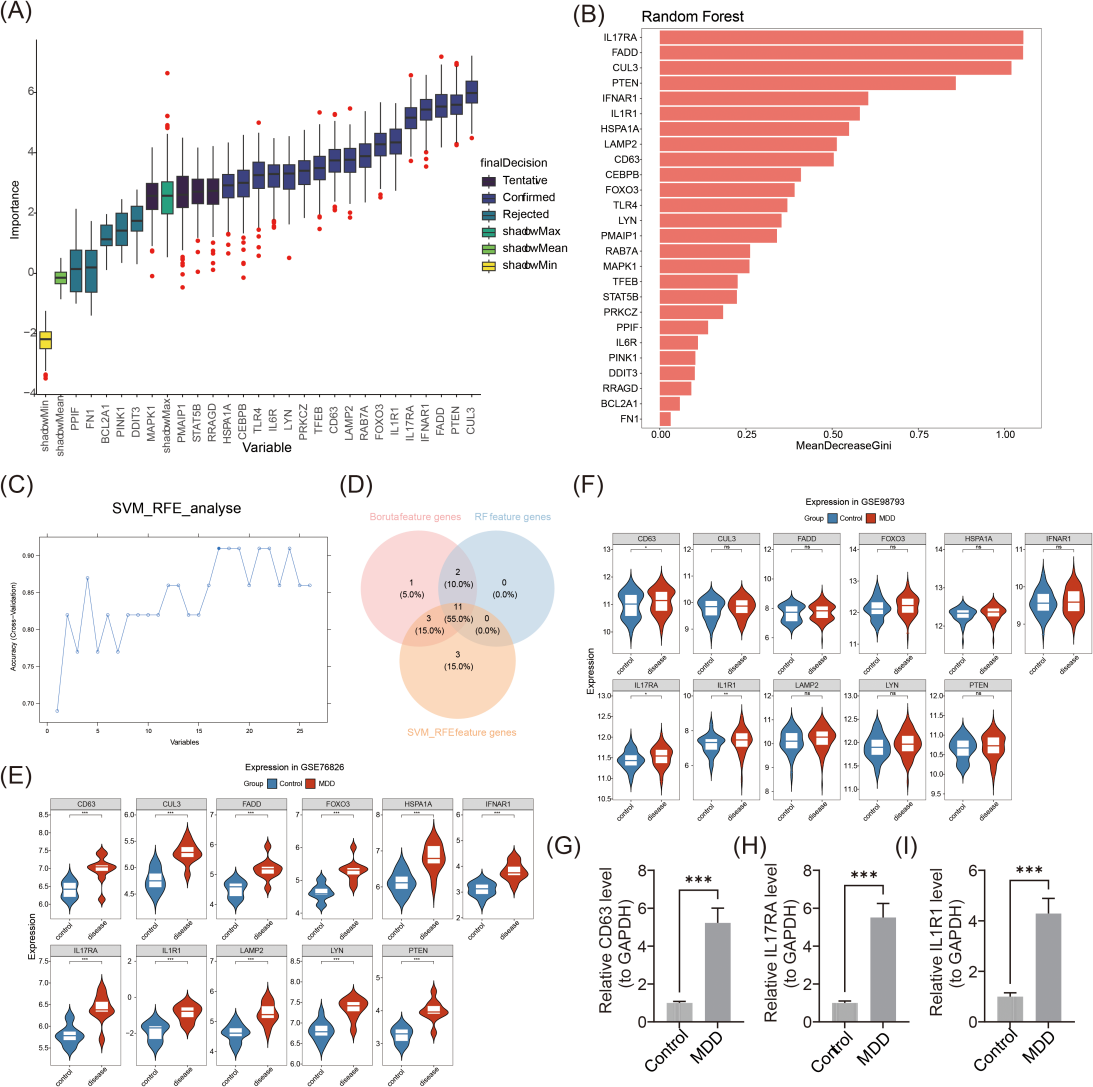
Identification and expression validation of biomarkers. **(A)** Boruta algorithm screening for signature genes. **(B)** Random forest model screening for key genes. Horizontal coordinates indicate immune cells and vertical coordinates indicate enrichment scores of immune cells. **(C)** Feature gene screening using the SVM-RFE algorithm. **(D)** Venn diagram of genetic intersections identified by the three algorithms. **(E)** Expression analysis of candidate biomarkers in GSE76826. ***p < 0.001. **(F)** Expression analysis of candidate biomarkers in GSE98793. *p < 0.05, **p < 0.01, ns: p > 0.05. **(G-I)** RT-qPCR results for CD63, IL17RA, and IL1R1. ***p < 0.001.

The Boruta algorithm robustly identified important features by comparing shadow features. From this analysis, 17 Boruta feature genes were selected (**Figure 3A**). RF analysis further identified 13 RF feature genes, including CEBPB, LYN, and CD63, with an importance value median greater than 0.3464709 (**Figure 3B**). SVM-RFE analysis revealed 17 SVM-RFE feature genes (**Figure 3C**). By intersecting the genes identified by the three algorithms, 11 candidate biomarkers were obtained: LYN, CD63, HSPA1A, IL17RA, CUL3, FOXO3, LAMP2, FADD, PTEN, IFNAR1, and IL1R1 (**Figure 3D**). Gene expression analysis further confirmed that CD63, IL17RA, and IL1R1 exhibited consistent expression patterns across the GSE76826 and GSE98793 datasets, with significant differences observed between MDD and control groups (p < 0.05) (**Figures 3E, F**). Specifically, CD63, IL17RA, and IL1R1 were markedly up-regulated in MDD compared to controls. Finally, the results of RT-qPCR demonstrated that CD63 (FC = 5.23), IL17RA (FC = 5.51), and IL1R1 (FC = 4. 29) were significantly highly expressed in MDD patients (p < 0.001). (**Figures 3G–I**). Overall, the expression trends of these biomarkers were consistent with those observed in the GSE76826 and GSE98793 datasets, supporting the reliability of the biomarkers identified through this filtering and characterization process.

### 3.4 The precise predictive capability of the nomogram

**Figure 4:**
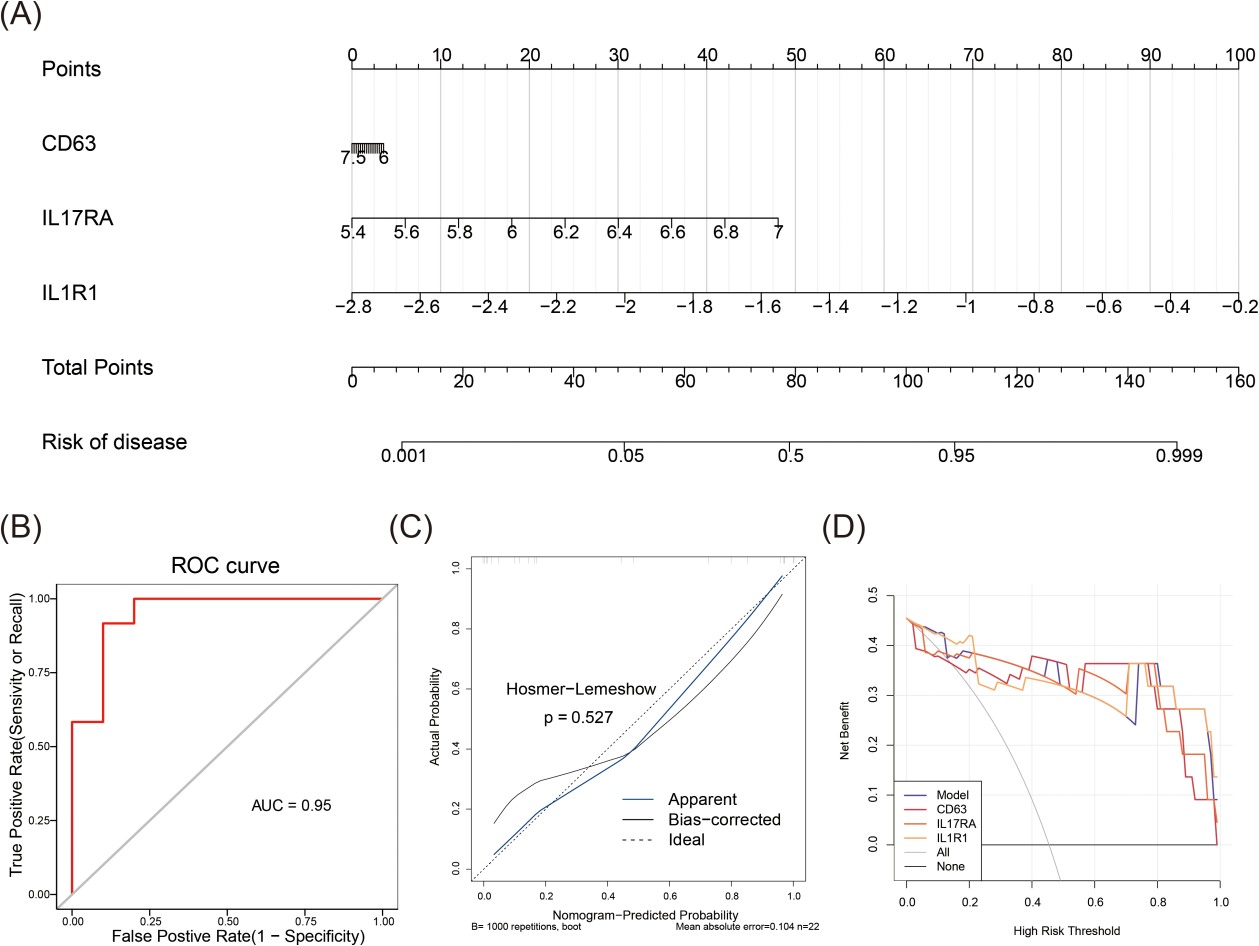
Predictive accuracy of the nomogram. **(A)** Nomogram predicting the accuracy of MDD. **(B)** ROC curve analysis for the nomogram. **(C)** Calibration curve for the nomogram. **(D)** Decision curve analysis (DCA) for the nomogram.

A nomogram was initially developed to evaluate the predictive accuracy of CD63, IL17RA, and IL1R1 for MDD (**Figure 4A**). The subsequent ROC curve analysis of the nomogram model revealed exceptional performance (AUC = 0.95) (**Figure 4B**). The calibration curve’s slope, nearing ideal, confirmed the model’s predictive accuracy (p = 0.527) (**Figure 4C**). The DCA curve further demonstrated the model’s robust clinical utility (**Figure 4D**). Overall, these results highlight the nomogram’s high predictive accuracy and substantial clinical applicability for MDD.

### 3.5 Immune infiltration analysis of biomarkers

**Figure 5:**
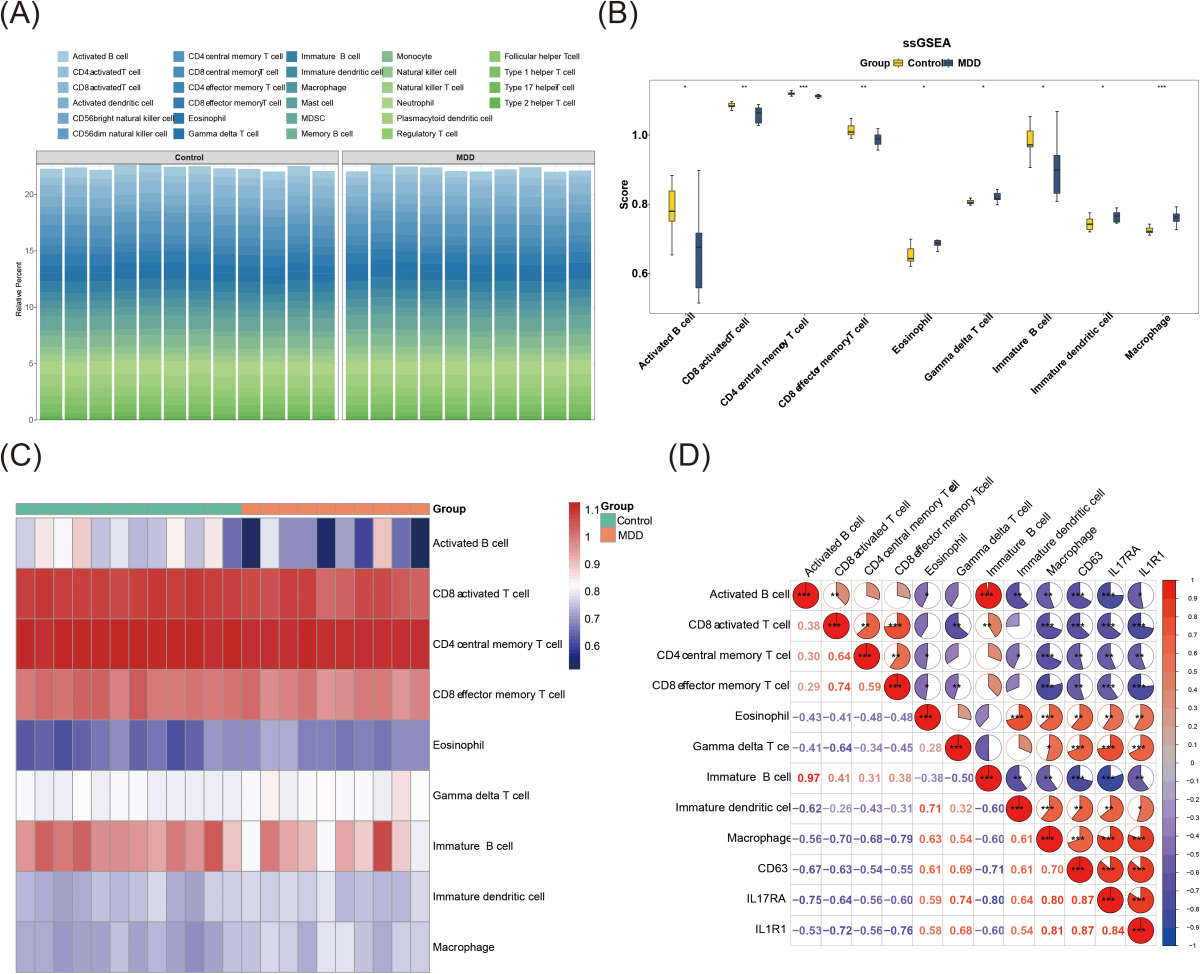
Immune infiltration analysis of biomarkers. **(A)** Estimation of the proportions of 28 different immune cell types in GSE76826 samples. **(B)** Differences in immune cell expression between the MDD and control groups in GSE76826. *p < 0.05, **p < 0.01, ***p < 0.001. **(C)** Heatmap of immune cell abundance in MDD and control groups, with red indicating high abundance and blue indicating low abundance. **(D)** Heatmap showing correlations between differential immune cells and biomarkers. Red indicates positive correlation, the stronger the correlation, the redder the color, blue indicates negative correlation, the stronger the correlation, the bluer the color, and the percentage of colors in the circle indicates the size of the correlation coefficient.

Immune cell presence and activity within the microenvironment significantly impact patient outcomes, making it a key area for therapeutic investigation. The estimated proportions of 28 distinct immune cell types across samples in GSE76826 are shown in **Figure 5A**. Analysis of immune cell infiltration revealed significant differences in the enrichment scores of nine cell types. A marked increase in eosinophils, gamma delta T cells, immature dendritic cells, and macrophages was observed in the MDD group, while activated B cells, CD8+ T cells, CD4+ central memory T cells, CD8+ effector memory T cells, and immature B cells showed a notable decrease (**Figure 5B**). The immune infiltration levels of these differential cell types in the control and MDD groups are displayed, with activated B cells exhibiting reduced expression in the MDD group (**Figure 5C**). These results suggest a distinct immunological landscape in MDD, potentially reflecting altered activation and maturation of critical immune cells.

Analysis of immune cell associations revealed the strongest positive correlation between activated B cells and immature B cells (cor = 0.97, p = 6.93 × 10^-17^), while the strongest negative correlation was observed between CD8 effector memory T cells and macrophages (cor = -0.79, p = 8.02 × 10^-5^) (**Figure 5D**, **Supplementary Table S10**). Notably, CD63 showed a strong positive correlation with macrophages (cor = 0.70, p = 5.89 × 10^-5^), while IL17RA (cor = 0.87, p = 1.03 × 10^-7^) and IL1R1 (cor = 0.87, p = 1.62 × 10^-7^) exhibited strong positive correlations with CD63. In contrast, CD63 (cor = -0.71, p = 8.02 × 10^-5^) and IL17RA (cor = -0.80, p = 3.27 × 10^-5^) demonstrated strong negative correlations with immature B cells, and IL1R1 (cor = -0.76, p = 2.30 × 10^-5^) negatively correlated with CD8 effector memory T cells.

### 3.6 Regulatory network analysis and drug prediction

**Figure 6:**
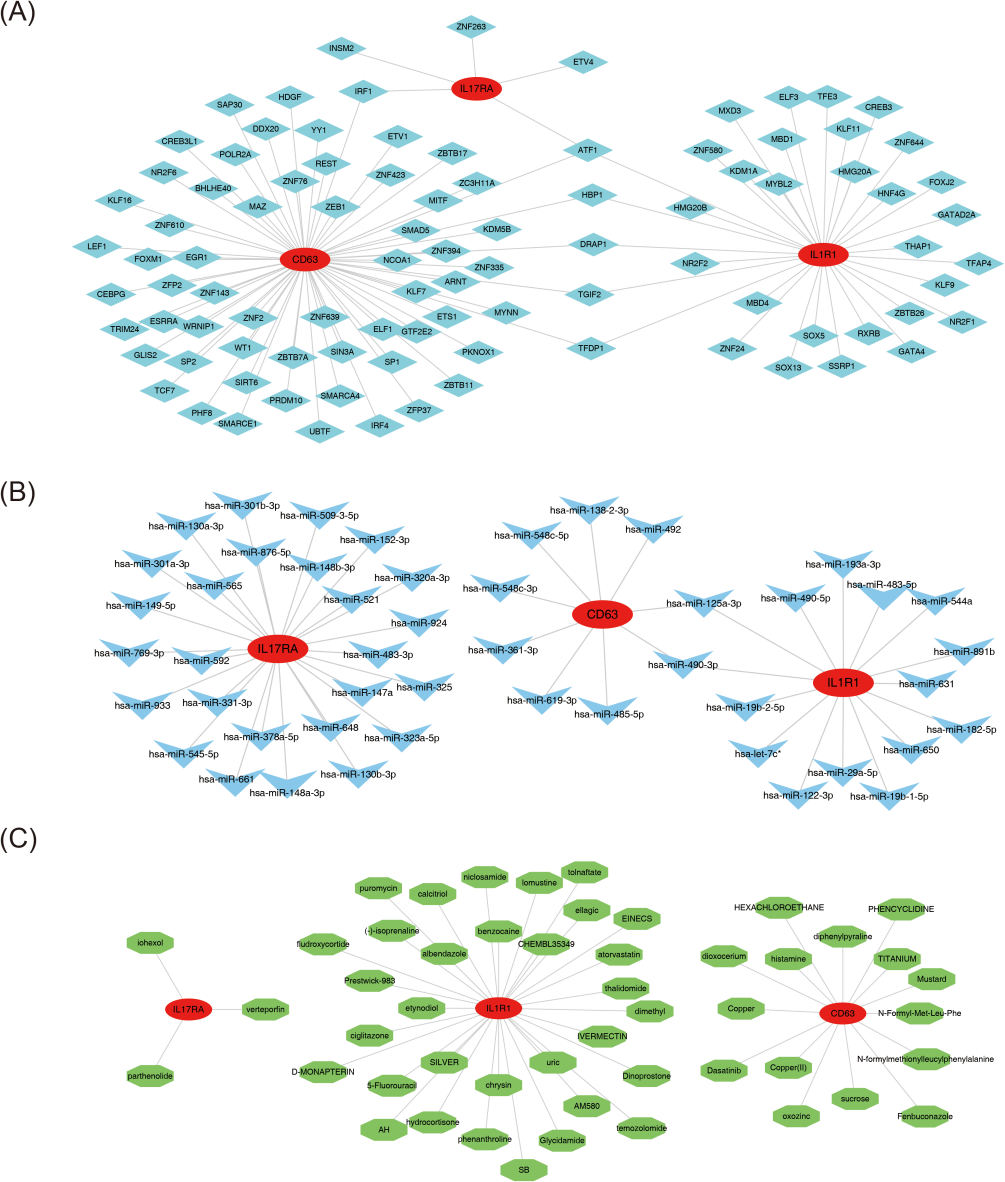
Regulatory network analysis and drug prediction. **(A)** Transcription factor (TF)-biomarker regulatory network. Red circles represent biomarkers, and blue diamonds represent transcription factors. **(B)** miRNA-biomarker (mRNA) regulatory network. Red circles represent biomarkers, and blue arrows represent miRNAs. **(C)** Drug-biomarker interaction predictive network. Red nodes represent biomarkers, and green nodes represent drugs.

Molecular regulatory networks are essential for understanding gene regulation mechanisms in biological cells and the pathogenesis of related diseases. The constructed TF-mRNA network identified 95 TFs predicted by the biomarkers (**Figure 6A**). ATF1 emerged as the common TF across all three biomarkers, while IRF1 was shared between CD63 and IL17RA, and HBP1, DRAP1, TGIF2, and TFDP1 were common to CD63 and IL1R1. The microcosm database predicted that CD63 targets 9 miRNAs, IL17RA targets 26, and IL1R1 targets 15. The miRNA-mRNA regulatory network revealed interactions such as hsa-miR-148b-3p-IL17RA, hsa-miR-548c-3p-CD63, and hsa-miR-490-5p-IL1R1 (**Figure 6B**). Notably, the shared miRNAs between CD63 and IL1R1 included hsa-miR-490-3p and hsa-miR-125a-3p. Furthermore, small-molecule drug therapies are crucial in managing MDD. Based on interaction scores, the top 50 potential drugs (scores ranging from 120.43 to 1,733.47) were identified for MDD. Fifteen agents (e.g., histamine, diphenylpyraline, and fenbuconazole) were linked to CD63, while IL17RA was associated with three drugs (verteporfin, parthenolide, and iohexol), and IL1R1 was connected to 32 drugs (e.g., etynodiol, ciglitazone, and chrysin) (**Figure 6C**). This approach emphasizes the potential for personalized therapies, enhancing understanding of drug responses in MDD and advancing precision psychiatry.

## 4 Discussion

The onset age and somatic symptoms of MDD are influenced by genetic factors, with different genetic variants contributing to significant variations in the age of onset, as well as the types and severity of somatic symptoms (44). Delving further into the biological basis of MDD may yield substantial clinical benefits. This study began with bioinformatics techniques, including differential expression, correlation, and algorithmic analysis based on protein-protein interactions, which led to the identification of 26 key candidate genes. Machine learning algorithms and gene expression validation subsequently confirmed three critical biomarkers for MDD: CD63, IL17RA, and IL1R1. A nomogram model was developed based on these biomarkers, demonstrating strong predictive performance. Additionally, immune infiltration analysis, molecular regulatory network analysis, and drug prediction were conducted, providing foundational support for pharmacological targeting, treatment, and diagnosis of MDD.

CD63, a tetraspanin protein present on various cell types, is particularly abundant on lysosomes and multivesicular bodies (45). It is involved in several cellular functions, including cell signaling, adhesion, and immune regulation (46, 47). A study examining type 2 diabetes mellitus (T2DM) found significantly higher CD63 expression in T2DM individuals with comorbid depression compared to those with diabetes alone (48), suggesting that CD63 could serve as a potential biomarker for depression, particularly in individuals with comorbid T2DM. Chronic stress, a well-known trigger for depression, has been shown to increase pro-inflammatory platelet activity, affecting brain function through inflammatory pathways and worsening depressive symptoms (49). In states of chronic stress, the number of CD63^+^ platelets notably rises, leading to elevated pro-inflammatory platelet activity in the bloodstream (50). These findings propose that increased CD63^+^ platelets may serve as an intermediary linking physical inflammatory conditions to psychosomatic health issues. Another study also demonstrated increased CD63 expression in platelets from patients with depression (51), suggesting that alterations in CD63 expression could be pivotal in platelet hyperactivation among depressed individuals. In this study, CD63 expression was significantly upregulated in patients with MDD, aligning with existing evidence that CD63 exacerbates the pathological course of depression. This finding offers novel insights into the diagnosis and treatment of depression, indicating that monitoring CD63 expression levels could be valuable for assessing patient conditions and evaluating treatment efficacy.

Interleukin 17 Receptor A (IL17RA), a cell surface receptor in the IL-17 receptor family, is primarily responsible for mediating IL-17 signaling, contributing to inflammatory processes and immune responses (52). Elevated IL-17 levels in the gut and serum are linked to chronic neuroinflammation, which reduces 5-HT concentrations in the hippocampus and induces depressive-like behaviors in mice (53). Furthermore, IL-17 has been identified as a specific inflammatory marker associated with Parkinson’s disease depression (54). An epigenetic meta-analysis on MDD suggests that IL17RA plays a role in the neurobiological mechanisms of depression, likely through inflammation (55). This study similarly designates IL17RA as a risk factor for MDD, supporting the widely accepted mechanism in which IL17RA modulates neural function through IL-17-mediated inflammatory responses. In addition, disruption of the IL-17A-IL-17RA interaction was found to broaden the inflammatory response, which had a significant effect on mitochondrial fission, modulating mitochondrial fission sensitivity by inhibiting phosphorylation of downstream Drp-1 through STAT-3 signaling (56), which may lead to mitochondrial dynamics dysfunction, thereby affecting the development and progression of depression (23). Consequently, targeting IL17RA emerges as a promising therapeutic strategy for MDD.

Interleukin 1 Receptor Type 1 (IL-1R1) is a receptor involved in the signaling of Interleukin 1 (IL-1), a cytokine secreted primarily by macrophages and monocytes, which plays a pivotal role in inflammatory and immune responses (57, 58). IL-1R1 expression is upregulated in the lymphocytes of patients with MDD, with its mRNA level in lymphocytes serving as a potential biomarker for depression (59). Genome-wide association studies (GWAS) have pinpointed specific single nucleotide polymorphisms (SNPs) in the IL1R1 gene, such as rs2540315 and rs75746675, that are linked to antidepressant responses (60). Inflammatory cytokines like IL-1, IL-2, and IL-6 have been shown to predict treatment outcomes in treatment-resistant depression (61). In this study, we found a potential link between CD63, IL17RA, IL-1R1, and MDD, all of which are associated with inflammation. Circulating mitochondrial DNA in MDD patients has been shown to contribute to inflammation in MDD (62), and further studies found that monocytes from patients who experienced childhood adversity had an enhanced inflammatory response, which would drive the cells toward pyroptosis (63). These findings suggest that inflammation, mitochondrial function and programmed cell death play important roles in the pathogenesis of MDD. However, further studies are needed to explore their specific mechanisms in MDD.

Immunoinfiltration analysis revealed an increased proportion of eosinophils, γδ T cells, and macrophages in patients with MDD. A recent study highlighted eosinophils as an independent predictor of MDD, playing a role in the pathological inflammatory response associated with the condition (64). γδ T cells, a type of unconventional T cell, have been shown to influence host susceptibility to chronic stress within the gut system (65), thereby raising the risk of depression. Additionally, substantial evidence supports macrophage activation in the central nervous system, where they release inflammatory cytokines that contribute to MDD progression (66). Correlation analysis of immune cell differences demonstrated a strong positive correlation between CD63 and macrophages, suggesting that elevated CD63 levels may interact synergistically with macrophage activation in MDD. Previous studies have indicated a reduction in mature B cells that are antigen-naive, immune-regulatory B cell subsets, and transitional B cells in patients with MDD (67). This study similarly found decreased expression of activated B cells and immature B cells in MDD, consistent with the general decline of B cell populations observed in the condition. The reduction in immature B cells may reflect issues with B cell generation and differentiation in patients with MDD, while the decrease in activated B cells could impair the maintenance of immune balance. The strong negative correlation between IL17RA and immature B cells suggests that targeting IL17RA or the IL-17 signaling pathway may aid in restoring immature B cell numbers and function, potentially alleviating MDD symptoms.

In this study, functional enrichment analysis revealed that targets such as CD63, IL17RA, and IL1R1 are involved in depression-related pathways, including the HIF-1, MAPK, and PI3K-Akt signaling pathways. Notably, the activation of the HIF-1 signaling pathway has been shown to mitigate depression progression in rats by enhancing synaptic function and neurogenesis (68). Current research identifies the HIF-1 pathway as a key therapeutic target for depression (69). Similarly, the MAPK signaling pathway plays a critical role in depression, with the MAPK-CREB1-BDNF signaling axis, a branch of the MAPK pathway, reducing depressive symptoms in mice by modulating hippocampal neuronal damage (70). In MDD, the MAPK pathway serves as a central hub in the pathological mechanisms of disease progression, with molecular alterations triggered by various stressors being regulated through this pathway (71). Moreover, the PI3K/Akt signaling pathway, essential for brain cell growth and survival, influences depression through several mechanisms, including modulation of neurotransmitter activity, regulation of neuroinflammation, promotion of hippocampal neurogenesis, and repair of synaptic damage (72). These findings suggest that these targets may modify depression’s pathological mechanisms by participating in or influencing these pathways.

PCR results aligned with bioinformatics analysis, showing upregulated expression of CD63, IL17RA, and IL1R1 in the MDD group compared to the control group, further confirming the reliability of the bioinformatics predictions. Through comprehensive molecular regulatory network analysis, transcription factors such as ATF1, IRF1, HBP1, DRAP1, TGIF2, and TFDP1 were identified as potential regulatory factors in depression, warranting further exploration. Additionally, the miRNA-mRNA regulatory network highlighted miRNAs like hsa-miR-148b-3p, hsa-miR-548c-3p, and hsa-miR-490-5p as valuable for depression-related research. Although several potential drugs targeting CD63, IL17RA, and IL1R1 were identified, limited research exists on their effects on MDD or depression. The development of targeted therapies for MDD, with a focus on clinical translation, remains a critical area for future research.

This study identified CD63, IL17RA, and IL1R1 as key targets in MDD and elucidated the potential mechanisms through which they exert their effects, laying a foundation for future MDD research. However, the study has certain limitations. First, the inclusion of additional samples is necessary to improve the reliability of the predicted biomarkers. Second, while bioinformatics analysis offers valuable insights, further research is needed to translate these findings into clinical applications. Finally, additional gene knockout and animal cell experiments should be conducted to further validate the biological functions of these genes.

## Data Availability

The datasets [ANALYZED] for this study can be found in the [GEO] [https://www.ncbi.nlm.nih.gov/geo/] with login numbers (GSE76826 and GSE98793), [MitoCarta3.0] [http://www.broadinstitute.org/mitocarta], [STRING] [https://string-db.org/], [microcosm] [https://tools4mirs.org/software/mirna_databases/microcosm-targets/] and [DSigDB] [https://dsigdb.tanlab.org/DSigDBv1.0/].

## Glossary

MDD: major depressive disorder
DEGs: differentially expressed genes
PCDs: programmed cell death-related genes
MitoGs: mitochondrial-related genes
cor: correlation coefficient
PPI: protein-protein interaction
RT-qPCR: reverse transcription-quantitative polymerase chain reaction
ROS: reactive oxygen species
PGC-1α: peroxisome proliferator-activated receptor gamma coactivator 1-α
PCD: programmed cell death
MitoGs-DEGs: mitochondrial-related differentially expressed genes
PCDs-DEGs: programmed cell death-related differentially expressed genes
GEO: gene expression omnibus
FC: fold change
GO: gene ontology
KEGG: kyoto encyclopedia of genes and genomes
STRING: search tool for the retrieval of interacting genes
MCC: maximum clique centrality
MNC: maximum neighborhood component
DMNC: density of maximum neighborhood component
RF: random forest
SVM-RFE: support vector machine-recursive feature elimination
ROC: receiver operating characteristic
AUC: area under the curve
DCA: decision curve analysis
TFs: transcription factors
miRNAs: microRNAs
DSigDB: drug signatures database
BPs: biological processes
CCs: cellular components
MFs: molecular functions
T2DM: type 2 diabetes mellitus
IL17RA: interleukin 17 receptor A
IL-1R1: interleukin 1 receptor type 1
IL-1: interleukin 1
GWAS: genome-wide association studies
SNPs: single nucleotide polymorphisms
