# Supplementary material for: Identification and experimental validation of biomarkers associated with mitochondrial and programmed cell death in major depressive disorder
Source: Front Psychiatry. 2025 Apr 30;16:1564380. doi: 10.3389/fpsyt.2025.1564380 (PMC12075303; doi:10.3389/fpsyt.2025.1564380)
Supplement: Supplementary file 1 [file DataSheet1.pdf]

![frontiers]

Supplementary Material

Supplementary Figures

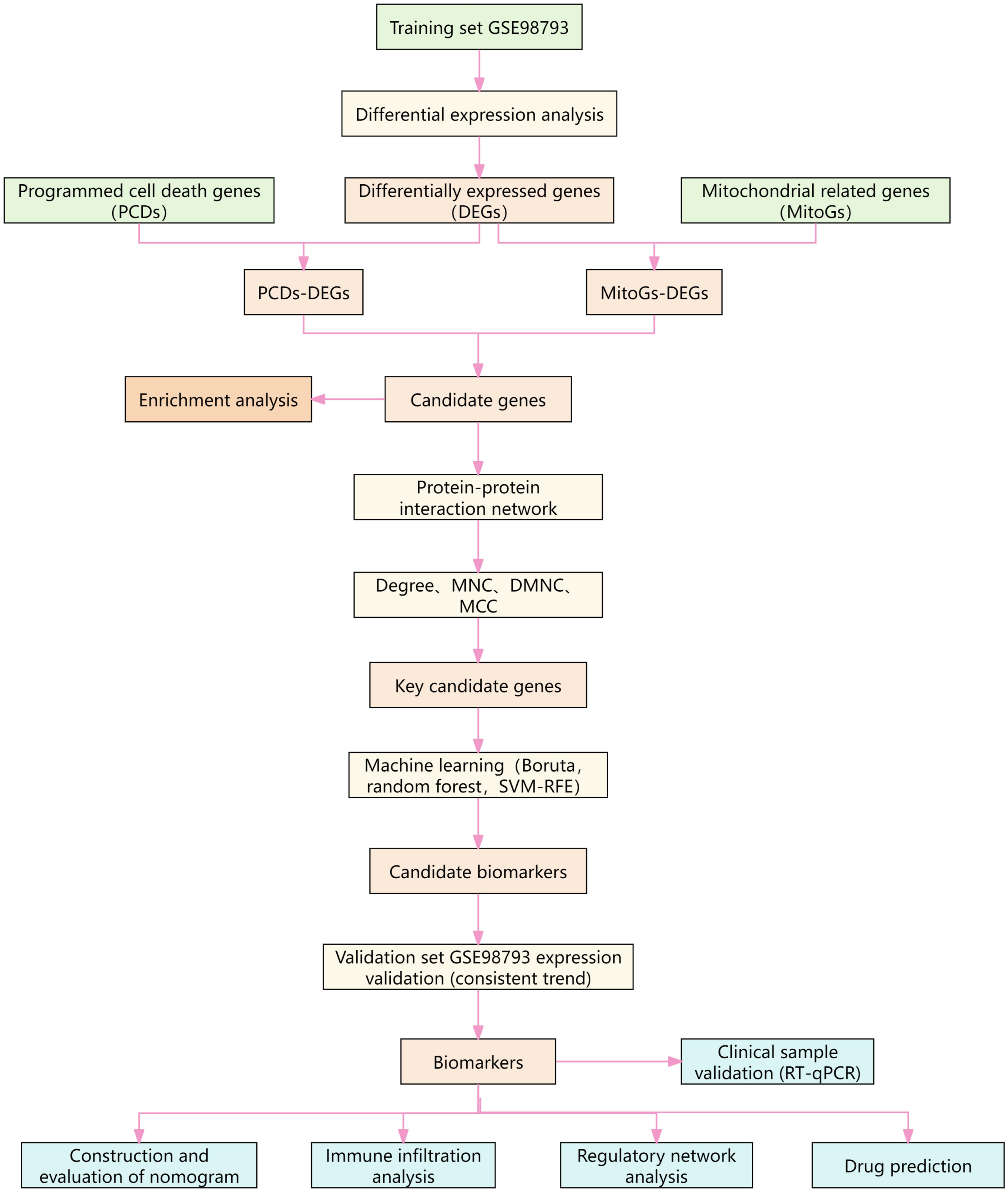

Supplementary Figure 1 Flowchart for identifying biomarkers associated with mitochondrial and programmed cell death in major depressive disorder.

Supplementary Tables

Supplementary Table 1. Clinical information for the dataset GSE76826.

Supplementary Table 2. Clinical information for the dataset GSE98793.

Supplementary Table 3. Information on 1,548 programmed cell death genes.

Supplementary Table 4. Information on 1,136 mitochondrial related genes.

Supplementary Table 5. The information of primer sequences for RT-qPCR.

Supplementary Table 6. Information on 55 candidate genes.

Supplementary Table 7. Results of GO enrichment analysis.

Supplementary Table 8. Results ofKEGG enrichment analysis.

Supplementary Table 9. Information on 26 key candidate genes.

Supplementary Table 10. Results of correlation analysis between differential immune cells.
